# The role of biotic interactions in shaping distributions and realised assemblages of species: implications for species distribution modelling

**DOI:** 10.1111/j.1469-185X.2012.00235.x

**Published:** 2012-06-12

**Authors:** Mary Susanne Wisz, Julien Pottier, W Daniel Kissling, Loïc Pellissier, Jonathan Lenoir, Christian F Damgaard, Carsten F Dormann, Mads C Forchhammer, John-Arvid Grytnes, Antoine Guisan, Risto K Heikkinen, Toke T Høye, Ingolf Kühn, Miska Luoto, Luigi Maiorano, Marie-Charlotte Nilsson, Signe Normand, Erik Öckinger, Niels M Schmidt, Mette Termansen, Allan Timmermann, David A Wardle, Peter Aastrup, Jens-Christian Svenning

**Affiliations:** 1 Department of Bioscience, Aarhus UniversityFrederiksborgvej 399, 4000 Roskilde, Denmark; 2 Greenland Climate Research Centre, Greenland Institute of Natural ResourcesPostboks 570, 3900 Nuuk, Greenland; 3 Faculty of Biology and Medicine, Department of Ecology and Evolution, University of LausanneLausanne, Switzerland; 4 Ecoinformatics and Biodiversity Group, Department of Bioscience, Aarhus UniversityAarhus, Denmark; 5 Department of Bioscience, Aarhus UniversityVejlsøvej 25, 8600 Silkeborg, Denmark; 6 Biometry and Environmental System Analysis, University of FreiburgTennenbacher Str. 4, 79104 Freiburg/Breisgau, Germany; 7 Department of Biology, University of BergenN-5020 Bergen, Norway; 8 Finnish Environment Institute, Natural Environment CentrePO Box 140, FIN-00251 Helsinki, Finland; 9 Department of Bioscience, Aarhus UniversityGrenåvej 14, 8410 Rønde, Denmark; 10 Department of Community Ecology, Helmholtz Centre for Environmental Research—UFZTheodor-Lieser-Str. 4, 06120 Halle, Germany; 11 Department of Geosciences and Geography, University of HelsinkiFIN-00014 Helsinki, Finland; 12 Department of Forest Ecology and Management, Swedish University of Agricultural SciencesUmeå 901 83, Sweden; 13 Dynamic Macroecology, Swiss Federal Research Institute WSLZürcherstr. 111, CH-8903 Birmensdorf, Switzerland; 14 Swedish University of Agricultural Sciences, Department of EcologyPO Box 7044, SE-75007 Uppsala, Sweden; 15 Department of Environmental Science, Aarhus UniversityFrederiksborgvej 399, 4000 Roskilde, Denmark; 16 Université de Picardie Jules Verne, Ecologie et Dynamiques des Systèmes Anthropisés1 Rue des Louvels, 80000 Amiens, France

**Keywords:** biotic interaction, climate, macroecology, prediction, sampling, scale, spatial extent, species distribution model, species assemblage

## Abstract

Predicting which species will occur together in the future, and where, remains one of the greatest challenges in ecology, and requires a sound understanding of how the abiotic and biotic environments interact with dispersal processes and history across scales. Biotic interactions and their dynamics influence species' relationships to climate, and this also has important implications for predicting future distributions of species. It is already well accepted that biotic interactions shape species' spatial distributions at local spatial extents, but the role of these interactions beyond local extents (e.g. 10 km^2^ to global extents) are usually dismissed as unimportant. In this review we consolidate evidence for how biotic interactions shape species distributions beyond local extents and review methods for integrating biotic interactions into species distribution modelling tools. Drawing upon evidence from contemporary and palaeoecological studies of individual species ranges, functional groups, and species richness patterns, we show that biotic interactions have clearly left their mark on species distributions and realised assemblages of species across all spatial extents. We demonstrate this with examples from within and across trophic groups. A range of species distribution modelling tools is available to quantify species environmental relationships and predict species occurrence, such as: (*i*) integrating pairwise dependencies, (*ii*) using integrative predictors, and (*iii*) hybridising species distribution models (SDMs) with dynamic models. These methods have typically only been applied to interacting pairs of species at a single time, require *a priori* ecological knowledge about which species interact, and due to data paucity must assume that biotic interactions are constant in space and time. To better inform the future development of these models across spatial scales, we call for accelerated collection of spatially and temporally explicit species data. Ideally, these data should be sampled to reflect variation in the underlying environment across large spatial extents, and at fine spatial resolution. Simplified ecosystems where there are relatively few interacting species and sometimes a wealth of existing ecosystem monitoring data (e.g. arctic, alpine or island habitats) offer settings where the development of modelling tools that account for biotic interactions may be less difficult than elsewhere.

## CONTENTS

- Introduction 16
- The role of biotic interactions in shaping species' spatial patterns 18
  1. Biotic interactions within the same trophic level 18
    - Animals 18
    - Plants 18
  2. Biotic interactions across trophic levels 20
    - Predator-prey 20
    - Animals and food plants 20
    - Interactions and feedbacks between plants and soil biota 21
    - Host-parasite and host-pathogen 21
- Accounting for biotic interactions in species distribution models (SDMs) 22
  1. Approaches 22
    - Integrating pairwise dependences 22
      - Multiple independent equations 22
      - Multiple simultaneous equations 23
    - Using surrogates for biotic-interaction gradients 23
    - Hybridizing SDMs with dynamic models 24
  2. Challenges common to these approaches 24
    - Inferring causation from spatial data 24
    - Species occur in complex networks 25
    - Multicollinearity 25
    - Biotic interactions are not constant in time and space 25
  3. Wanted: fine-grained biotic data along environmental gradients over large spatial extents 25
  4. Conclusions 26
  5. Acknowlegements 26
  6. References 27

## I. INTRODUCTION

**Fig. 1:**
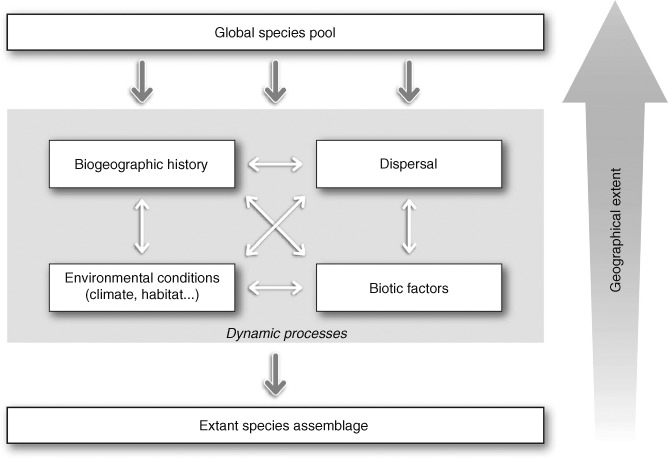
The main processes and filters that interact to structure species assemblages across geographical extents. Refined from Lortie *et al.* (2004) and Guisan & Thuiller (2005).

Which species will occur together in the future, where, and why? Addressing these questions is a challenging task in the face of global change, because species have distinct responses to changes in the environment that depend on complex relationships to their ecological attributes, such as abiotic tolerances, dispersal capacity, history, and biotic interactions, that each vary in time and space (e.g. Lortie *et al.*, 2004; Guisan & Thuiller, 2005; Ferrier & Guisan, 2006; Algar *et al.*, 2009) (Fig. 1). Moreover, novel communities may emerge from individualistic range dynamics (Williams & Jackson, 2007; Algar *et al.*, 2009; Stralberg *et al.*, 2009; Schweiger *et al.*, 2010).

Biotic interactions are known to affect species' spatial patterns *via* several mechanisms, such as predation, competition, resource-consumer interactions, host-parasite interactions, mutualism and facilitation (Bascompte, 2009; van Dam, 2009). Many examples from the ecological literature document that these interactions are not static in space and time and can be linked with the impacts of changing climate in many complicated ways (Forchhammer *et al.*, 2005; Braschler & Hill, 2007; Suttle, Thomsen & Power, 2007; Tylianakis *et al.*, 2008; Gilman *et al.*, 2010). In a recent state-of-the-art review of community interactions under climate change, Gilman *et al.* (2010) argued that interactions among species can strongly influence how climate change affects species at every scale and that failure to incorporate these interactions limits our ability to predict species responses to climate change. Moreover, a synthesis of 688 published studies (Tylianakis *et al.*, 2008) revealed substantial variability in both the magnitude and direction of effects of global change drivers on any type of biotic interaction. A large body of literature has also documented that biotic interactions can affect species response to abiotic environmental changes differently along environmental gradients, and that abiotic environmental changes can likewise influence the nature of biotic interactions (Brooker & Callaghan, 1998; Davis *et al.*, 1998; Choler, Michalet & Callaway, 2001; Callaway *et al.*, 2002*a*; Brooker, 2006; Meier *et al.*, 2010*a*). Thus, there is a pressing need to advance methods to account for the dynamics and complexity of biotic interactions in future predictions.

**Fig. 2:**
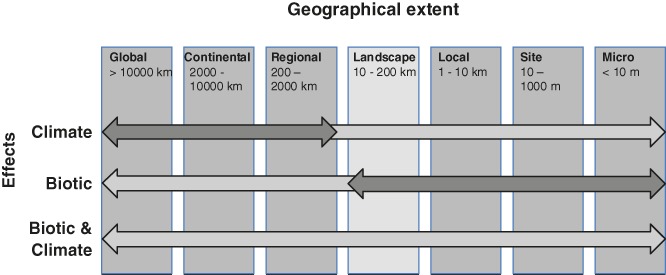
Spatial extents at which the influence of environmental variables is likely to be detectable in spatially explicit data. Dark arrows are those presented in Pearson & Dawson (2003), and these are updated with examples cited herein (light arrows).

Evidence of how biotic interactions have shaped species distributions at broad spatial extents (e.g. landscapes, regions, continents and beyond) has received relatively little attention in the context of predicting future species assemblages, and likewise, their role in shaping patterns at these scales has been largely dismissed as unimportant. Pearson & Dawson (2003) proposed a conceptual framework in which biotic interactions are expected to play a role in shaping species distributions only over local extents while other factors, such as climate, play a role at broader spatial extents, but not more locally. The framework proposed by Pearson & Dawson (Fig. 2) is drawn from a tradition followed by a number of ecologists (e.g. Whittaker, 1975) who concluded that the distribution of the terrestrial biomes of the world could be explained by the distribution of mean temperature and precipitation values alone. Moreover, in a recent review, Wiens (2011) concluded that there is a paucity of good examples of large-scale patterns created by biotic interactions. They speculated that this could be either because few studies have explored this directly, or that biotic interactions only rarely play a role in broad-scale spatial patterns. Jablonski (2008) also called for an in- tegrative approach that aimed to incorporate biotic interactions into explanations of broad-scale ecological and evolutionary changes, despite the fact that many of these interactions are relatively ephemeral and local in nature. Brooker *et al.* (2009) in reply to Ricklefs (2008) highlighted the need for studying biogeographic processes across a range of temporal and spatial scales, and postulated that biotic interactions can play a potential role at all scales, although they are decreasingly influential at regional and continental scales. Soberón (2007) argued for the distinction between the Grinnellian class of niche, defined by certain abiotic variables typically available at coarse resolution and broad spatial extents (e.g. average temperature, precipitation, etc.), and the Eltonian class of niche, defined by variables representing biotic interactions and resource-consumer dynamics and typically measured at local scales. Soberón (2007) pointed out that the spatial structure of variables defining Grinellian and Eltonian niches is a largely unexplored research area, and the degree of spatial structure will probably depend on the organisms being considered.

Evidence for how biotic interactions shape species distributions beyond local extents can be found in contemporary and palaeoecological studies of individual species ranges, functional groups, and species richness patterns (e.g. Maran & Henttonen, 1995; MacFadden, 2006; Kissling, Rahbek & Böhning-Gaese, 2007; and many more examples below). The members of a given local community are constrained by the regional species pool, which itself does not only depend on the cumulative effects of local processes happening within the region, including biotic interactions, but also on processes operating over broader extents, such as speciation, historical regional extinctions, and regional immigration (Ricklefs, 1987, 2008; Ricklefs & Schluter, 1993; Zobel, 1997; Brooker *et al.*, 2009). Documenting the role of biotic interactions in shaping spatial patterns across spatial extents is therefore an important step towards understanding and accounting for them in predictions of future species assemblages.

In recent decades, species distribution models (SDMs) have been widely applied to model and predict the potential distributions of species in response to global change. These methods typically relate species distributions or abundance to spatially explicit abiotic constraints (e.g. climate, land use) (Guisan & Zimmermann, 2000; Guisan & Thuiller, 2005) with recent advances that incorporate mechanisms such as dispersal (e.g. Engler & Guisan, 2009) and biotic interactions, to make the models more realistic. Among these emergent advances, methods dealing with biotic interactions (Araújo & Luoto, 2007; Heikkinen *et al.*, 2007; Schweiger *et al.*, 2008,2011; Meier *et al.*, 2010b; Pellissier *et al.*, 2012) present the greatest challenges.

Herein, we review the role of biotic interactions in shaping the spatial distributions of species, with a detailed and novel focus on the broadest (regional to global) spatial extents, where biotic interactions have been argued to be unimportant (Willis & Whittaker, 2002; Pearson & Dawson, 2003). We then highlight emerging methods to quantify relationships among interacting species from spatially explicit data, and to account for them in SDMs. By accounting for biotic interactions we mean adjusting species' predictive probabilities of occurrence based on spatial patterns in certain other species at a given time. Inferring the identities of particular species interactions at a given place (predation, competition, etc.) cannot be done with statistical correlative methods of spatially explicit data without supplementation from other lines of evidence, and we therefore do not address this issue. For our purposes here we focus our discussion on interspecific interactions and distinguish between biotic interactions within the same trophic level and across trophic levels. Grain (also known as resolution) and extent are among the most commonly used attributes when referring to scale. We refer to fine or coarse resolution (e.g. 100 m resolution is finer than 1 km resolution). We distinguish resolution from geographical extent, which can range anywhere from broad to narrow in focus (e.g. continental extent *versus* a local extent, respectively).

## II. THE ROLE OF BIOTIC INTERACTIONS IN SHAPING SPECIES' SPATIAL PATTERNS

In the context of spatial ecology, biotic interactions have been generally dismissed as unimportant beyond the local scale (Fig. 2). Nevertheless, biotic interactions have left their mark on species' distributions and realized assemblages of species, with effects evident across spatial scales, as supported by multiple lines of evidence. Below, we examine examples of this evidence separately for predator-prey dynamics, animal competition, and plant-plant, plant-animal, plant-soil, and host-parasite/pathogen interactions to address how they have each shaped spatial patterns beyond the local scale.

### (1) Biotic interactions within the same trophic level

#### (a) Animals

**Fig. 3:**
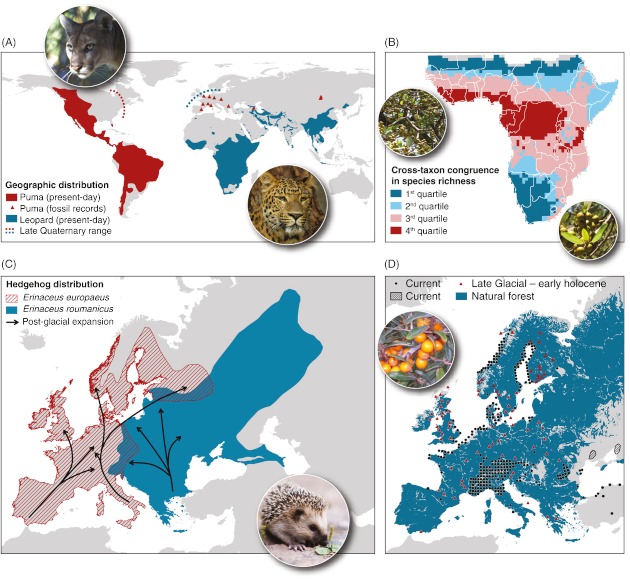
Examples of how biotic interactions can shape distributions and realized assemblages of species at broad spatial scales. (A) Present-day geographic ranges of pumas (*Puma concolor*, red) in the New World and leopards (*Panthera pardus*, blue) in the Old World (IUCN, 2010) plotted together with additional range extent of both species during the Late Quaternary (dotted curved lines) and palaeorecords of pumas (*Puma pardoides*, triangles) from Eurasia (based on Hemmer *et al.*, 2004). Expansion of leopards during the early Middle Pleistocene probably replaced pumas in their Old World area of origin (Hemmer, 2004). (B) Cross-taxon congruence in species richness of figs (*Ficus* spp.) and avian obligate frugivores across sub-Saharan Africa (data from Kissling *et al.*, 2007). Species richness of both groups was subdivided into quartiles with cells in dark red (fourth quartile) indicating areas where both groups have highest species richness and cells in dark blue (first^t^ quartile) indicating lowest richness. The example illustrates how the broad-scale distribution of consumers is linked to the distribution of keystone food plants. (C) Distribution of hedgehogs (*Erinaceus* spp.) in Europe (IUCN, 2010) with post-glacial expansion routes (black arrows) as proposed by Hewitt (1999). Mutual exclusion over large spatial extents of such closely related species is likely to result from competition or other negative interactions. (D) Present-day geographic range (black circles) and Late Glacial-Early Holocene pollen records (red triangles) of *Hippophaë rhamnodies* compared with the current natural distribution of forest in Europe (blue shading). Natural post-last glacial maximum (LGM) reforestation of central and northern Europe restricted this shade-intolerant shrub *H. rhamnoides* to marginal tree-less habitats in the region. The current distribution is represented by diagonal shading. Present and past occurrence records were compiled from Lang (1994) and Hultén & Fries (1986). The distribution of natural forest in Europe was based on Bohn & Neuhäusl (2003).

Competition between animals can affect range limits and geographic diversity patterns. In desert rodents in the American Southwest, geographic ranges overlap much less than expected in similar-sized granivorous species, while this is not the case when broader size ranges or feeding guilds are considered (Bowers & Brown, 1982). The largely allopatric ranges of African equids have been partially attributed to competitive exclusion (Bauer, McMorrow & Yalden, 1994). There are also a number of striking cases involving mutual exclusion over large spatial extents of closely related species and subspecies along hybrid zones which could be attributed to competition, e.g. *Erinaceus europaeus* and *E. roumanicus* (Santucci, Emerson & Hewitt, 1998) (Fig. 3) and *Mus musculus* and *M. domesticus* (Boursot *et al.*, 1993; Mitchell-Jones *et al.*, 1999; Ganem, Litel & Lenormand, 2008). That competitive interactions may affect geographic ranges is also illustrated by displacements of native species by other closely related introduced species. A famous example is the displacement of European red squirrel *Sciurus vulgaris* by the introduced *S. carolinensis* across much of the British Isles (Bertolino, 2008), although this negative interaction could at least partially involve pathogen-mediated apparent competition (see Section II.2*d*). Another case is the involvement of the invasive North American mink *Mustela vison* in the cross-continental range collapse of the European mink *M. lutreola* (Maran & Henttonen, 1995). The above examples all concern mammals, but evidence also comes from invertebrates such as insects and arachnids (Reitz & Trumble, 2002).

The palaeorecord provides examples of taxon replacements at broad spatial extents that are highly suggestive of competitive exclusion. Some of these examples concern long-term (10^7^–10^8^ years), large-scale expansion of one large phylogenetic clade at the expense of another. Classical cases are the global decline of the chthamaloid barnacles and the simultaneous expansion of balanoid barnacles (Stanley & Newman, 1980) and the Great American Faunal Interchange, which led to the decline or complete extinction of a number of South American mammal groups with the advent of ecologically equivalent groups from North America (Webb, 1976, 2006; MacFadden, 2006). Another case is the early Tertiary extinction of pleasiapiform primates and multituberculates, hypothesized to be due to competitive exclusion from diversifying rodents (Maas, Krause & Strait, 1988). Other examples are provided by mammalian carnivores, e.g. pumas (*Puma pardoides*) during the early Middle Pleistocene being replaced in their Old World area of origin by expanding leopards (*Panthera pardus*) (Fig. 3A) (Hemmer, Kahlke & Vekua, 2004). The Pliocene-Pleistocene expansion of true elephants from Africa across Eurasia and partially into North America and the broadly synchronous regional extinctions of gomphotheres and mammutid mastodonts could also be listed here (Agustí & Antón, 2002; Morgan & Lucas, 2003; Vislobokova, 2005). There are also many cases of species replacements within genera, e.g. repeated mammal species invasions of Europe from Asia-Africa with complete extinction of local forms, or their partial extinction with survival only in geographically isolated areas (‘competitive refugia’) (Randi, 2007), a controversial example being the range contraction of neanderthals (*Homo neanderthalensis*) in the face of modern humans (*Homo sapiens*) (Mellars, 2004).

#### (b) Plants

Evidence of how plant competition and facilitation affect species' geographic ranges over broad geographic extents is limited and often indirect, perhaps reflecting that competitive exclusion between pairs of plant species may be much less efficient due to their sessile nature and contingent highly localized competitive interactions relative to in mobile organism groups. In a recent meta-analysis, Götzenberger *et al.* (2012) found no evidence that competition has shaped plant distributions at broad spatial extents, but also concluded that the data were insufficient to test this based on their analysis of the meta-data from 91 articles. Further research is needed in this area to provide conclusive evidence. One example that may suggest competition at broad spatial scales is a study of two congeneric heathland shrub species (genus *Ulex*) which show strong negative associations at three spatial scales of investigation (Bullock *et al.*, 2000). The authors suggest that over the ranges of these *Ulex* species competitive superiority is probably determined by climate, whereas at range margins other physical factors (e.g. soil) might drive the outcome of competition. The ability of some plants to grow and reproduce in botanical gardens under other climatic conditions has also been interpreted to suggest that biotic interactions restrict their natural distributions (Vetaas, 2002), although the role of other factors in such studies often cannot be fully disentangled from the biotic interaction signal. Specific life forms in plants can potentially influence the species ranges of other life forms. For instance, species-specific facilitative interactions among vascular epiphytes and trees (Callaway *et al.*, 2002b) suggest that trees' geographic ranges might strongly influence epiphytes' geographic ranges. Further facilitative effects among plants have been implied in the diversification of modern ferns which seem to have benefited from the more complex canopy structure of angiosperm trees relative to gymnosperms (Schneider *et al.*, 2004; Schuettpelz & Pryer, 2009). For competitive interactions, pollen records indicate that the natural reforestation of central and northern Europe after the postglacial period probably forced the shade-intolerant shrub *Hippophaë rhamnoides* (Fig. 3D) to contract its formerly large geographic range to marginal tree less habitats in this region (Bartish, Kadereit & Comes, 2006). Competition has also been proposed to have a role in shaping communities as inferred from pollen records of forest succession during Quaternary interglacials, where light-demanding pioneer shrub and dwarf-shrub species were first replaced by light-demanding and taller pioneer trees and then by shade-tolerant late-successional tree species (Bennett & Lamb, 1988). Studies of local interactions between *Artemisia tridentata* and *Pinus ponderosa* present competition as a process contributing to post-Pleistocene replacement of conifer forests in the Great Basin with desert shrubs (Callaway *et al.*, 1996). Other examples involving disturbances come from grasses which can exclude trees from dry regions by causing an increase in fire frequency (Bond, Midgley & Woodward, 2003). Moreover, during the shift from the wet Tertiary period to the dry Quaternary that saw the development of most global deserts, evidence from palaeobotanical, ecological, and phylogenetic analyses, support the hypothesis that a large number of ancient Tertiary species in Mediterranean-climate ecosystems persisted through facilitative or “nurse” effects with modern Quaternary species (Valiente-Banuet *et al.*, 2006).

### (2) Biotic interactions across trophic levels

#### (a) Predator-prey

The presence of predators, particularly large top predators (apex consumers), can strongly influence the abundance, distribution and range limits of prey species in terrestrial, fresh water and marine systems (Estes *et al.*, 2011). For instance, in a controlled experiment in Northwestern Canada and Alaska it was shown that wolf (*Canis lupus*) predation is the main factor limiting recruitment of caribou (*Rangifer tarandus*) and moose (*Alces alces*), and survival of adult moose (Hayes *et al.*, 2003). Similarly, the eradication of wolves and grizzly bears (*Ursus arctos*) in the Grand Teton National Park resulted in a strong increase in moose density, with consequent overbrowsing of riparian vegetation and the disappearance of migratory birds in the impacted willow communities (Berger *et al.*, 2001). Important examples of the potentially strong spatial effects of predators on prey come from the introduction of mammalian predators to islands. For instance, the introduction of mammalian predators to New Zealand has caused a number of local and global extinctions (Bellingham *et al.*, 2010). Particularly devastating have been the introductions of rats (*Rattus spp*.) which are the largest contributors to seabird extinction and endangerment worldwide (Jones *et al.*, 2008). Towards the margins of a species distribution, the mortality inflicted by a predator can create abrupt range edges of a focal prey species (Holt & Barfield, 2009). Another mechanism by which top predators might influence species distributions is by controlling mid-sized predators, or mesopredators. In Australia, introduced mid-sized predators (the red fox, *Vulpes vulpes,* and the feral cat, *Felis catus*) have caused strong declines and extinctions of native small marsupials, and the persecution and resulting rarity of Australia's largest native predator, the dingo (*Canis lupus dingo*), might have played a critical role in allowing mid-sized predators to overwhelm marsupial prey, triggering extinction over much of the continent (Johnson, Isaac & Fisher, 2007). Interestingly, specialist predators can potentially also facilitate larger prey ranges due to e.g. behavioural escape mechanisms (Holt & Barfield, 2009).

#### (b) Animals and food plants

The dependence of animals on plants can be an important determinant of species distributions at broad spatial extents. Comparisons of folivorous insects on temperate and tropical tree species of comparable phylogenetic distribution show that similar numbers of folivorous insect species can coexist in both regions (Novotny *et al.*, 2006). Together with findings that food resources are not more finely partitioned among folivorous insects in tropical than in temperate forests, these results suggest that the latitudinal gradient in insect species richness could be a direct function of plant diversity (Novotny *et al.*, 2006). Similarly, the distribution and species richness of frugivorous birds across Africa has been shown to be spatially linked with the diversity of keystone food plants (i.e. figs, genus *Ficus*) (Kissling *et al.*, 2007) (Fig. 3B). In the Neotropics, the species richness and abundance of nectarivorous hummingbirds at a geographic scale is strongly associated with the seasonal abundance of flowers but weakly with environmental factors (Abrahamczyk & Kessler, 2010). In plant-pollinator systems, it has even been demonstrated that specific floral characters of food plants can determine visitor restriction suggesting that functional traits of food plants can determine the distribution of nectarivorous consumers along climatic gradients (Dalsgaard *et al.*, 2009). For granivorous species, a study on the acorn woodpecker (*Melanerpes formicivorus*) in the Southwestern United States and along the Pacific coast has found that the distributional limit of this bird species is set by sites where the diversity of oaks (genera *Quercus* and *Lithocarpus*) drops to a single species and not by the limit to the distribution of oaks *per se* (Koenig & Haydock, 1999). Some species-specific evidence on the importance of food plants as range determinants has also been provided by studies on butterflies and their larval host plants. For instance, current range expansion of a polyphagous butterfly in Britain is associated with the exploitation of more widespread host plants suggesting that polyphagy may enhance the ability of species to track climate change (Braschler & Hill, 2007). Overall, there is good evidence that food plants can be a limiting factor for the distribution of animal consumers, but there is also potential for the opposite, i.e. that broad-scale distributions or range limits of plants are constrained by the distribution of animals. For example, transplant experiments of *Arnica montana*, have revealed that slug herbivory limits the lower elevational range of this subalpine plant (Bruelheide & Scheidel, 1999). Also, the colonization of islands by fleshy-fruited plants provides evidence that range limits and distributional areas of these species strongly depend on animal dispersers (Thornton, Compton & Wilson, 1996; Shanahan *et al.*, 2001).

#### (c) Interactions and feedbacks between plants and soil biota

The interactions between plants and their associated soil biota can serve as an important determinant of plant range expansion under global change. As such, there is some evidence that range-expanding plant species may escape antagonistic soil biota (van Grunsven *et al.*, 2010), consistent with the ‘enemy release hypothesis' (Keane & Crawley, 2002). Further, there is evidence that plant species which are undergoing active range expansion, for example as a result of climate change, show different interactions with their below-ground and above-ground antagonists than related species that do not. In a study of six congeneric pairs of range-expanding and non-expanding plants in the Netherlands, Engelkes *et al.* (2008) showed that non-expanders were consistently strongly negatively influenced by soil biota (presumably soil pathogens) and by a generalist herbivore, whereas the range expanders were only weakly negatively or neutrally affected. The obvious implication of this is that some plant species cannot undergo range expansion because they are kept in check by antagonistic biotic interactions, while others can undergo range expansion because they are less adversely affected. Further evidence emerges from the plant invasion literature; it has been recognized in a growing number of studies that invasive and non-invasive plant species differ in their interactions with soil antagonists (Klironomos, 2002; van der Putten, Klironomos & Wardle, 2007). As such, species that rapidly undergo range expansion during invasion have a demonstrated capacity to escape their soil antagonists and thus negative plant-pathogen interactions; examples include rapid range expansion of *Prunus serotina* in Europe (Reinhart *et al.*, 2003) and *Centaurea maculosa* in North America (Callaway *et al.*, 2004). Finally, range expansion of plants can also be influenced by interactions with their below-ground mutualists. For example, range expansion of plant species can be greatly impaired if compatible mycorrhizal fungi are absent from the area that they are expanding into, and there is evidence of expansion of *Pinus* species being regulated by the presence *versus* absence of appropriate strains of mycorrhizal fungi (Richardson *et al.*, 2000; Nunez, Horton & Simberloff, 2009). It has thus been hypothesized that postglacial migration rates of some tree species may have been constrained by the slow migration of their mycorrhizal mutualists (Wilkinson, 1998). Similarly the ingress of nitrogen-fixing plants (i.e. those that undergo mutualistic interactions with bacteria that convert atmospheric N into plant-available forms) to new habitats could at least partly and in specific cases be regulated by whether or not appropriate strains of symbiotic bacteria are present in the new habitat (Richardson *et al.*, 2000; van der Putten *et al.*, 2007).

#### (d) Host-parasite and host-pathogen

The presence and distribution of parasites and pathogens can potentially be a driver of diversity and distribution patterns from regional to global extents (Ricklefs, 2010*a*, *b*). A widely cited example of how pathogens can affect the distribution of host species is the introduction of the North American grey squirrel *Sciurus carolinensis* which has replaced the native red squirrel *S. vulgaris* over much of the UK. The emergence of a novel parapoxvirus that is lethal to red squirrels but seemingly harmless to grey squirrels has probably contributed to this process (Tompkins, White & Boots, 2003). A similar case involves the decline and partial extinction of the native noble crayfish *Astacus astacus* in many European lakes which was probably driven by the fungal pathogen *Aphanomyces astaci* introduced to Europe with the North American signal crayfish *Pacifastacus leniusculus* (Josefsson & Andersson, 2001). Similarly, the distribution of many bird species across islands in the Caribbean cannot be readily linked to dispersal limitation or competition for resources and it has hence been suggested that some distribution anomalies could be explained by the presence of pathogens (Ricklefs, 2010b). In terms of parasites, the population declines of grey partridges *Perdix perdix* in the UK have been attributed to parasite-mediated apparent competition with the ring-necked pheasant *Phasianus colchicus via* a shared parasitic nematode (Tompkins, Draycott & Hudson, 2000*a*; Tompkins *et al.*, 2000b). In neotropical montane frogs, chytrid fungus infections have been found to explain the extinction or critical endangerment of 72 of the 85 species of the bufonid genus *Antelopus* (Skerratt *et al.*, 2007). Further to this, chestnut blight caused by the introduction of the fungus *Cryphonectria parasitica* was responsible for the loss of American chestnut trees (*Castanea dentate*) over large parts of their range in North America (Paillet, 2002). The aphid-like pest, hemlock woolly adelgid, which parasitizes eastern and Carolina hemlock (*Tsuga canadensis* and *caroliniana*) and can kill trees within 4–5 years, has caused marked reductions in hemlock populations in the eastern United States (Lovett *et al.*, 2006). A study of how light availability converts an endosymbiotic fungus to a pathogen that influences seedling survival a tropical palm (*Iriartea deltoidea*) furthermore provides mechanistic evidence that fungal pathogens may shape plants' realized niche and distribution (Álvarez-Loayza *et al.*, 2011). Fresh water snails were found to be more susceptible to pathogens or predation at the range margin where they encountered greater physiological stress (Briers, 2003).These examples suggest that host-parasite and host-pathogen interactions can play an important role for species distributions at broad spatial scales.

## III. ACCOUNTING FOR BIOTIC INTERACTIONS IN SPECIES DISTRIBUTION MODELS (SDMS)

Having established that biotic interactions have potentially important implications for shaping species distributional patterns across spatial extents, we can now turn to the challenge of how to account for these interactions in spatially explicit modelling tools. Theoretically, species distribution models (SDMs) are based on the concept of realized niche (*sensu*Hutchinson, 1957; Pulliam, 2000), and a number of studies suggest that they do not completely account for biotic interactions (see Zimmermann *et al.*, 2010, for a short review); recent findings call for the development of tools that more appropriately explicitly and comprehensively account for these (Leathwick & Austin, 2001; Meier *et al.*, 2010*a*, *b*; Pellissier *et al.*, 2012). This represents an important challenge but at the same time a fundamental opportunity to bring more ecological theory into SDMs (Austin, 2002).

Here, we propose possible ways to account for biotic interactions in SDMs by reviewing and discussing the most promising and up-to-date methods. In particular, we will consider the many opportunities offered by community ecology and population biology as conceptual toolboxes that could help in accounting for interactions among species. We restrict our discussion to interspecific interactions, particularly any influence one species might have on one or several others in modulating the relationships these species have to their environment. We highlight the strengths and weakness of the approaches that have been attempted to date, and also present alternative or complementary approaches that deserve further consideration as novel modelling frameworks.

### (1) Approaches

#### (a) Integrating pairwise dependences

When considering a given set of species in a given area, biotic interactions are expected to generate two main results: (*i*) to influence species-environmental relationships; (*ii*) to produce a non-random pattern of species co-occurrence. To date, patterns of species co-occurrence have been used to integrate biotic interactions into empirical models of species distribution. In an SDM framework, the most straightforward approach has been to use the distribution pattern of one species and a suite of abiotic predictors to predict one or several other species (Guisan, Weiss & Weiss, 1999; Leathwick & Austin, 2001; Araújo & Luoto, 2007; Heikkinen *et al.*, 2007; Pellissier *et al.*, 2010, 2012); results have shown that the predictive power of SDM models increased with inclusion of these biotic predictors. This kind of approach has been increasingly used and examples include predictions of species interacting as predator and prey (e.g. Redfern *et al.*, 2006), animal and food plants (Preston *et al.*, 2008; Schweiger *et al.*, 2011, 2008), in resource competition (e.g. Meier *et al.*, 2010b; Pellissier *et al.*, 2012), in mutualism (e.g. Gutierrez *et al.*, 2005) and facilitation (e.g. Heikkinen *et al.*, 2007). In these SDM examples accounting for biotic interactions, the ecological links between pairs of species are known *a priori*, and usually supported by multiple lines of evidence (e.g. host-consumer relationships among some butterflies and host plants are well understood, and might be supported by behavioural or trophic studies, etc.). In cases where it is not known which species interact, this must be inferred from the data, ideally while accounting for geographic and environmental variation. Relatively few attempts have tried to infer which species interact from statistical correlations of patterns of co-occurrence in the species' and environmental data (Hawkins & Porter, 2003; path models: Kissling *et al.*, 2007; using residual co-variance: Ovaskainen, Hottola & Siitonen, 2010; using null-models of co-occurrences: Peres-Neto, Olden & Jackson, 2001). No method has been used both to detect interactions and subsequently to predict distributions.

To date, two general systems of multivariate regressions have been proposed to account for dependencies between pairs of species in a regional species pool. One system uses multiple independent equations (one equation for each species in the system) to relate each species to the abiotic part of its environment, and then subsequently explores the biotic relationships in the residuals of the equations (e.g. Peres-Neto *et al.*, 2001). The other system includes both abiotic and biotic factors in a series of interdependent, multivariate equations that are solved for simultaneously in simultaneous equation models.

##### (i) Multiple independent equations

In a multivariate regression context, the response variable for each species is modelled considering only environmental variables, and then speies interactions are accounted for as components of the residuals of the *independent* regression models solving for e.g. ***Y***_1,_***Y***_2,…_***Y***_*n*_ :


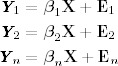
(1)

With **E**_*i*_∼MVN(0, **C**), where ***β***_*i*_**X** represents the matrix products for environmental predictors and where the vector of errors **E**_*i*_ follows a multivariate normal (MVN) distribution with **C** representing correlations between pairs of species, given the environmental predictors **X** included in the models.

Ovaskainen *et al.* (2010) provided a clear example of such an approach as multivariate logistic regression for modelling the community composition of wood-decaying fungal species for a total of 22 species in Finland. Sebastian-Gonzalez *et al.* (2010) adopted the same approach for modelling community composition of waterbird species breeding in artificial irrigation ponds using a total of seven species. In both cases, the authors included species interactions into **E**_*i*_ with **C** (in Equation 1) representing a correlation matrix describing whether each species pair co-occurs on the same sampling unit more or less often than expected by chance, after adjusting for the effect of the abiotic environment. Such an approach allows the detection of potential signals of biotic interactions between species pairs by analysing the correlation structure of the residuals after accounting for species-specific habitat requirements (Ovaskainen *et al.*, 2010).

Although the examples presented in Ovaskainen *et al.* (2010) and Sebastian-Gonzalez *et al.* (2010) account for spatially explicit environmental predictors and species' occurrences, they do not use information about the neighbourhood surrounding observation points, which may contain important ecological information shaped by relationships between interacting species. Spatial neighbourhoods in species occurrence data have been included in SDM models of single species (without considering biotic interactions) (Augustin, Mugglestone & Buckland, 1996). It is however, also possible to find evidence supporting interactions among species by considering the spatial structure of the residuals in a single species' model, although this by itself is not enough to indicate the presence of biotic interactions (Dormann *et al.*, 2007). The method requires fitting multiple spatial processes (one per species) and linking them using cross-covariance analyses of associations between species (Latimer *et al.*, 2009) in so called ‘multivariate spatial models'. Using data on four interacting species, Latimer *et al.* (2009) presented a linear model of coregionalization. In this multivariate spatial approach, the vector of errors **E**_*i*_ (in Equation 1) represents a spatial weights matrix where **C** is a covariance matrix of spatial association between species. In this case, the regression model accounts for environmental effects on a species' occurrence probability, while the two regressions are coupled through correlation (positive or negative) of their spatial errors. Thus unlike other methods described above, the parameter estimates in each regression account for those derived from abiotic environmental predictors, biotic predictors, and spatial structure in the species data. Nevertheless, it is important to point out that although the above methods can be used to detect the presence of biotic interactions, they cannot be used for prediction.

##### (ii) Multiple simultaneous equations

Unlike the methods presented above, which require fitting a series of independently solved regression equations, simultaneous equation modelling theoretically represents a more elegant approach in which the distribution of each species accounts for the influence of all other species at the same time, and interactions between species need not be considered separately. The idea, which has its roots in the field of econometrics (Greene, 1993), is to set a system of models where a series of equations are fitted simultaneously, and set with exogenous variables (independent variables that only occur on the right-hand side of any equation) and endogenous variables (dependent variables representing values of species abundance or occurrence and occur on either side of the equation).


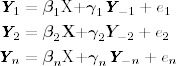
(2)

where ***β***_*i*_**X** represents the matrix products for environmental predictors and ***γ***_*i*_***Y***_−*i*_ represents the matrix products for all other **Y** responses but the *i*^th^ one, and *e*_*i*_ represents an error term. With this approach, each fitted response (e.g. ***Y***_1_, ***Y***_2_, ***Y***_*n*_) is included as an additional predictor in all other equations until equilibrium is reached across equations in an iterative process. This approach can be simplified based on ecological theory to define *a priori* which species are allowed to interact in equations or which species should be represented only in, for example, unidirectional interactions. Simultaneous equation models are a generic group of models that includes structural equation models. Although structural equation models have been used widely in ecology (Malaeb, Summers & Pugesek, 2000; e.g. Shipley, 2011), we are aware of no examples where simultaneous equation models have been used to model distributions of individual interacting species together.

#### (b) Using surrogates for biotic-interaction gradients

In the majority of ecological systems, disentangling all interactions between pairs of species may be almost impossible. One solution is to use SDMs in concert with surrogate variables that reflect spatial turnover or gradients in the distribution of biotic interactions in the landscape. Examples of these could be estimates of habitat productivity (from any empirical or process-based models of biomass, or retrieved from remote-sensing technologies), macroecological data, or macroecological predictions estimating species richness. Such methods require some sort of *a priori* ecological knowledge about the nature of the biotic interactions that are likely to be relevant (e.g. competition for light in the case of plant-plant interactions) and to identify the functional groups that are relevant to consider in models and to parameterise them. For example, in plants one may expect that the relative intensity of, for example, competition may vary along a productivity gradient (Callaway *et al.*, 2002*a*; Michalet *et al.*, 2006; Maestre *et al.*, 2010), and variables reflecting local productivity levels such as vegetation height or biomass can be included as surrogates of the intensity of biotic interactions (Midgley *et al.*, 2010). It can also be possible to set more proximal predictors to reflect interactions. For example, Meier *et al.* (2010*a*) built species distribution models for tree species including a biotic predictor that reflects competition for light, as defined by the cumulative leaf area index of all large trees within a stand.

Species richness patterns have been hypothesised to be under the influence of biotic interactions (Michalet *et al.*, 2006), among other drivers such as large-scale evolutionary forces and dispersal (Zobel & Pärtel, 2008). Based on the idea that species richness in a given unit should be limited and determined by macroecological factors (energy, heterogeneity, stability, etc.), a recent approach (SESAM) combines the use of macroecological models of species richness with stacked species distribution models (Guisan & Rahbek, 2011). In this method, macroecological models of species richness (e.g. Rahbek *et al.*, 2007) are used as a surrogate for the maximum number of species potentially co-occurring in pixels across a landscape, while a parallel step uses stacks of species distribution models to predict which species are likely to contribute to richness in a given pixel. Species richness estimates per pixel are then used to restrict the pool of species within relevant functional groups predicted to occur by SDMs. To illustrate this approach with an example, for a given study area, one would derive SDMs for each species within a particular functional group (e.g. species within a trophic position), and in parallel derive a species richness model for this target group of species. If the number of species predicted to occur in a pixel in the study area by SDMs exceeds the amount (*N*) predicted by the species richness model, only the (*N*) species predicted to have the highest probabilities of occurrence by the SDMs would be expected to occur in that pixel. Another refinement could draw upon species' functional traits.

For instance, methods have been developed to predict species abundances in plant communities from functional traits at the community level (Shipley, Vile & Garnier, 2006) and functional similarity between potentially coexisting species (Mouillot, Mason & Wilson, 2007). Integrating these approaches with SDMs remains to be explored but is theoretically very promising (McGill *et al.*, 2007).

#### (c) Hybridizing SDMs with dynamic models

In recent years there have been major advances to make SDMs more mechanistic and realistic by incorporating elements of process-based models (Thuiller *et al.*, 2008; Gallien *et al.*, 2010). Some examples of these include combining SDMs with dispersal models (e.g. Engler & Guisan, 2009; Smolik *et al.*, 2010), accounting for population dynamics, life-history traits and dispersal (e.g. Keith *et al.*, 2008), or modelling the dynamic nature of plant phenology in SDMs (e.g. Phenofit; Morin & Chuine, 2005). Such ‘hybrid models' show tremendous scope for modelling biotic interactions by accounting for changes in the availability and locations of suitable habitat along with mechanisms that govern which species have the possibility to interact with each other depending on their distinct possibilities to track changes in the landscape and colonise new areas. Integrating these mechanisms into SDMs is an important first step in attempting to overcome the unrealistic assumption inherent to the other modelling approaches described above, that biotic interactions are stable in space and time. Hybrid approaches can, in theory, be integrated with any of the approaches described above. However, a major drawback to hybrid approaches is the large number of parameters that need to be empirically estimated or assumed; notably, detailed information about species life history, dispersal, or demographics required to parameterise these models is often unavailable.

BIOMOVE is one example of a hybrid model that explicitly tries to account for biotic interactions. It allows annual simulations of plant species range shifts in response to changes in climate, habitat structure and disturbance (Midgley *et al.*, 2010). BIOMOVE integrates species' bioclimatic suitability and population-level demographic rates (using matrix calculation) with simulation of landscape-level processes including dispersal, disturbance, and species' response to dynamic dominant vegetation structure (Midgley *et al.*, 2010). In BIOMOVE, biotic interactions are mainly taken into account through resource competition determined by plant functional types (PFTs), this response to environmental changes is also modelled annually through dispersal, inter-PFT competition and demographic shifts (Midgley *et al.*, 2010). BIOMOVE is a relatively recent integration of individual-based models with SDMs, but examples of hybrid models can also be found in the forestry literature (Lischke *et al.*, 2006; Rüger *et al.*, 2007).

### (2) Challenges common to these approaches

#### (a) Inferring causation from spatial data

All the modelling approaches designed to account for biotic interactions described above have an important limitation. If the distribution of one species is shown to be highly dependent on the distribution of another species none of the approaches above can differentiate if this is due to a real biotic interaction between the two species or is better explained by one or more overlooked environmental factors not accounted for in the model. For example, consider the extreme case of two plant species: the aquatic waterlily (*Nymphea alba*) and the terrestrial couch grass (*Agropyron repens*). Using a model including only climatic predictors it is likely that the results show some residual negative dependence between the two species, that in the absence of any other information might be mistaken for evidence for competition. However, it is obvious in this case that these two species cannot occupy the same habitat (one is aquatic and the other not) so that they could never co-occur locally and ultimately never interact. Therefore, significantly positive or negative residual associations in the correlation matrix do not necessarily involve biotic interactions since important missing environmental variables might also be responsible. This is further complicated if the methods used to fit models affect the correlations that are calculated. For example, concerns that correlations can be influenced by how the absences are drawn to fit presence-absence models led Schweiger *et al.* (2008, 2011) to use only the range of the host plant species to select absences for calibrating their models of dependent butterfly species. All these pairwise approaches thus need some prior knowledge on the ecology of the species under study to parameterise the model—or a system of multiple models—including the appropriate environmental predictors at appropriate resolution, in order to avoid a high risk of type I error (i.e. concluding there is competitive exclusion when this is not the case).

#### (b) Species occur in complex networks

Species do not only interact in pairs, but can do so in complex networks (e.g. Bascompte, 2009). Higher-order interactions (Case & Bender, 1981; Billick & Case, 1994; Wootton, 1994) may lead to non-additive effects (Dormann & Roxburgh, 2005) which cannot be represented by the pairwise approaches presented above. If we consider two interacting species, A and B, we can also add a third component, species C, which may affect the way A interacts with B. A typical example of this situation is the case of a predator that shifts between two prey species or a pollinator that shifts between two or more host plants. However, such an interaction has not yet been investigated in an SDM framework.

#### (c) Multicollinearity

As for any classical regression technique, multicollinearity between predictors should be controlled, particularly when setting a system of equations (such as those interacting in simultaneous equation models), to avoid variance inflation of regression coefficients. Indeed, we may expect high correlation between some of the environmental predictors in X and species included as predictors Y_−i_, which may be a critical issue for the implementation of such approaches. Rigorous theoretical and empirical testing of these approaches in the context of SDMs is thus needed.

#### (d) Biotic interactions are not constant in time and space

All the predictive methods described herein are grounded in static correlations, and do not account for changes in biotic interactions in space and time. This is a critical assumption (though admittedly a necessary simplification as we begin to address the problem) that is likely to be challenged for many ecosystems or for particular organisms as we measure and quantify interactions. When SDM models are projected to pre-defined scenarios to reflect changes to the species pool (e.g. setting some relevant species' coefficients to zero) the predictions can be expected to be to at least some degree unrealistic merely because species' relationships to the abiotic and biotic environment can change with the species' pool. A large body of literature has also documented that biotic interactions can affect species response differently along environmental gradients (Brooker & Callaghan, 1998; Davis *et al.*, 1998; Choler *et al.*, 2001; Callaway *et al.*, 2002*a*; Brooker, 2006; Meier *et al.*, 2010*a*). For instance, Choler *et al.* (2001) found that the strength of interactions of particular alpine plant species with their neighbours generally depended on the species' position along the main environmental gradients. Quantifying changes in how much each species influences the spatial patterns of another species over environmental and geographic space is an important step in improving realism in these models. A first approach would simply include interaction terms between environmental predictors with the occurrence of an *a priori* defined potentially interacting species or functional group, or any surrogate of biotic interactions into the statistical models. A second and more comprehensive approach was proposed by Damgaard & Fayolle (2010) for quantifying the strength of competition in a pair of plant species as they changed over an environmental gradient (herbicide gradient). They proposed, if a measure of the ecological success of a species *i* is measured at variable densities of other species, **d**, and levels of one or more environmental gradients, **x**. Then the expected ecological success of an individual of species *i* may be expressed by an empirical function, *F*_*i*_(**d**,**x**); and a relative measure of the effect of biotic interaction may be defined as:


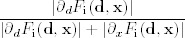
(3)

where |*∂*_*x*_*F*_i_(**d**,**x**)| is the absolute change in the ecological success of species *i* by changing the level of one abiotic factor (*x*), and |*∂*_*d*_*F*_i_(**d**,**x**)| is the change in the ecological success of species *i* by changing the density of one of the interacting species. Currently, methods do not exist to integrate such an approach into an SDM predictive framework.

## IV. WANTED: FINE-GRAINED BIOTIC DATA ALONG ENVIRONMENTAL GRADIENTS OVER LARGE SPATIAL EXTENTS

The species that will be encountered differ from location to location, and there is a need for replication in geographic and environmental space (defined by abiotic and biotic variables) to quantify and predict the outcome of biotic interactions. To increase our understanding of how local interactions affect broad-scale species distributions, there is a clear need to combine the information gained from fine-grained experimental and observational studies across a broad range of scales (Soberón, 2007; Brooker *et al.*, 2009). Such information should ideally reflect variation in biotic and abiotic environmental gradients across broad spatial extents. In order to improve our predictions on how species respond to a shifting environmental gradient, it is important that data are drawn from within the entire environmental space over which a species occurs. This is because data from part of the range might incorrectly estimate the relationship of the species to the environmental parameters e.g. by suggesting a linear relationship when a quadratic relationship might be more appropriate. Experiments covering large environmental gradients (Callaway *et al.*, 2002*a*; van der Putten *et al.*, 2007; van der Putten, Macel & Visser, 2010), especially that cover large spatial extents (continental and global) at fine spatial and temporal resolutions are needed to give insight into how the nature of biotic interactions change across environmental and geographical spaces.

Not only is there a dearth of experimental studies investigating biotic interactions and their effects on species distributions across large geographic extents, but those few observational studies that have included biotic interactions at broad spatial extents have used coarse-grained data (Hawkins & Porter, 2003; Araújo & Luoto, 2007; Heikkinen *et al.*, 2007; Kissling *et al.*, 2007; Schweiger *et al.*, 2011). Soberón (2007) pointed out that due to limitations in the data available, it is typically harder to measure the role of biotic interactions at broader spatial extents and resolutions than it is to assess the role of other variables (e.g. temperature, precipitation) at these resolutions. A paradigm shift is required towards collecting fine-grained experimental and observational data across large spatial extents stratified to represent variation in environmental gradients (e.g. Hirzel & Guisan, 2002) to better investigate the effect of biotic interactions on species distribution. Such cross-scale analyses have been proposed as one of the most productive avenues for future research to understand the role of local-scale processes such as biotic interactions in shaping large-scale processes such as species distribution (Brooker *et al.*, 2009).

An useful advances include the increasing amount of fine-grained species co-occurrence data collated in different databases. Fine-grained species co-occurrence, stored as species by sites matrices, consist of localities that are generally visited at least once and where all species detected were recorded at a specific time to yield presence-absence or abundance-dominance data. For plants, many electronic databases of vegetation plots (fine-grained species co-occurrence data) have been established in different European countries together containing >1800000 vegetation plots (Schaminee *et al.*, 2009). The emerging International Arctic Vegetation Database will represent the first vegetation database containing georeferenced plant data to encompass an entire global biome (Walker & Raynolds, 2011). Worldwide, the newly established Global Index of Vegetation-Plot Databases (GIVD), available online at http://www.givd.info/, gives an estimate of >2400000 vegetation plots with co-occurrence data stored in 132 databases from around the world (Dengler *et al.*, 2011). Together, these data already cover a large spatial and environmental extent, even after selecting vegetation plots that have been collected in a comparable way. Fine-grained monitoring surveys across large spatial extents observing fine-scale changes in co-occurrences with environmental shifts over time have huge potential in our context. Several such efforts have been established at regional extents, but some examples of monitoring surveys are now emerging across even larger spatial extents. A prominent example here is the Global Observation Research Initiative in Alpine environments (GLORIA) (http://www.gloria.ac.at/), monitoring 135 mountain summits across 36 regions for alpine plants.

In short, we call for observational data at high spatial resolution (fine-grained species co-occurrence data) with of high accuracy (GPS coordinates) compiled across large spatial extents. These data combined with experimental and long-term monitoring data will provide a better mechanistic understanding of how biotic interactions affect broad-scale species distributions, which in turn will make our predictions for future changes in biodiversity more accurate. In addition, such data might also be used directly in predictive modelling providing parameters for the model as suggested in the previous chapter. To sharpen the tools for modelling biotic interactions, it might be easier to begin with relatively simplified ecosystems where there are fewer interacting species to consider (e.g. arctic, alpine and islands). Moreover, researchers in these systems can already call upon a wealth of existing data (Callaghan *et al.*, 2004) from detailed population and ecosystem monitoring, including long time series of the dynamics of populations and communities.

## V. CONCLUSIONS

(1) An important step towards improving our capacity to predict future species assemblages from the rapidly increasing wealth of spatial data is to clarify the role of biotic interactions across spatial scales. Biotic interactions generally have been thought to be unimportant in determining large-scale distributions, but our review shows that they have left their mark on species distributions and realised assemblages of species at regional, continental and global extents. This conclusion is supported by numerous examples and multiple lines of evidence including experimental ecology, population ecology, community ecology, trophic ecology, comparative morphology, and palaeobiology.

(2) A variety of approaches is emerging to account for biotic interactions among species in distribution models. These include integrating pair-wise dependencies, using surrogates of biotic interaction gradients, consideration of functional groups, and use of hybrid approaches. Challenges common to these approaches include inferring causation from spatial data, overcoming multicollinearity, overcoming the complexity of species interaction networks, spatial and temporal variation in biotic interactions, and data paucity. Perspectives for refining predictions of SDMs by accounting for biotic interactions remain in the early stages of development. Their theoretical foundations, technical feasibility and utility with real data must be assessed.

(3) Our review demonstrates the need for temporally and spatially explicit species' data, sampled in strata that reflect environmental gradients across large spatial extents. This will help efforts to accurately quantify how biotic interactions influence species assemblages and the processes that shape them. Continued efforts to compile existing data across spatial extents and the inclusion of long-term monitoring data show great promise for informing methods to advance predictive modelling tools. Combined with the integration of diverse branches of ecology (e.g. species distribution modelling, population ecology and functional ecology) such efforts will facilitate achievable progress in predicting future species assemblages and distributions.

## VI. ACKNOWLEGEMENTS

This work originated in connection with a workshop for the TFI Networks ‘Effect Studies and Adaptation to Climate Change’ under Norforsk intiative (2011–2014). The working group is led by C. Damgaard. M.S.W. received support from the Greenland Climate Research Centre project number 6505, and The Research Council of Norway and the Directorate for Nature Management. This work was further supported by the Villum Kahn Rasmussen Foundation (grant VKR09b-141 to JCS) and the Danish Council for Independent Research–Natural Sciences (grant 10-085056 to SN). Support was also received from the European Commission (FP6-ECOCHANGE project) and Swiss National Science Foundation (BIOASSEMBLE project). This manuscript was greatly improved by comments from two referees.
